# Targeting HCV Entry For Development of Therapeutics

**DOI:** 10.3390/v2081718

**Published:** 2010-08-18

**Authors:** Flossie Wong-Staal, Andrew J. Syder, Jeffrey F. McKelvy

**Affiliations:** iTherX Pharmaceuticals, In., P.O. Box 910530, CA 910530, USA; E-Mails: dsyder@itherx.com (A.J.S.); jmckelvy@itherx.com (J.F.M.)

**Keywords:** HCV entry inhibitors, SR-BI, HCVpp, therapeutics development

## Abstract

Recent progress in defining the molecular mechanisms of Hepatitis C Virus (HCV) entry affords the opportunity to exploit new viral and host targets for therapeutic intervention. Entry inhibitors would limit the expansion of the infected cell reservoir, and would complement the many replication inhibitors now under development. The current model for the pathway of entry involves the initial docking of the virus onto the cell surface through interactions of virion envelope and associated low density lipoproteins (LDL) with cell surface glycosaminoglycans and lipoprotein receptors, followed by more specific utilization with other hepatocyte membrane proteins: Scavenger Receptor Class B type 1 (SR-BI), CD81, Claudin 1 (CLDN1) and Occludin (OCLN). The use of blockers of these interactions, e.g. specific antibodies, suggests that inhibition of any one step in the entry pathway can inhibit infection. Despite this knowledge base, the tools for compound screening, HCV pseudoparticles (HCVpp) and cell culture virus (HCVcc), and the ability to adapt them to industrial use are only recently available and as a result drug discovery initiatives are in their infancy. Several therapies aiming at modulating the virus envelope to prevent host cell binding are in early clinical testing. The first test case for blocking a cellular co-receptor is an SR-BI modulator. ITX 5061, an orally active small molecule, targets SR-BI and has shown potent antiviral activity against HCVpp and HCVcc. ITX 5061 has exhibited good safety in previous clinical studies, and is being evaluated in the clinic in chronic HCV patients and patients undergoing liver transplantation. Entry inhibitors promise to be valuable players in the future development of curative therapy against HCV.

## 1. Introduction

Hepatitis C virus (HCV) infection is a global disease of high prevalence. Remarkably, there is a single treatment option – a combination of subcutaneous α-interferon and oral ribavirin – which, at least in part due to intolerability and contraindication issues, provides effective treatment to only a minority of patients at the community level (as opposed to the clinical trial population) [1]. The ability to more effectively treat HCV has awaited scientific advances which enable targeted drug discovery. These have come in the form of the replicon system, facilitating the screening of compounds that inhibit viral replication [2], and the pseudotyped virus assay (HCVpp) [3,4], which allows screening of drugs that specifically inhibit entry of viruses bearing the HCV envelope proteins. More recent cloning of HCV capable of a complete replication cycle *in vitro* [5–7] has been valuable in validating drugs derived from the surrogate screening systems, and should also allow screening for inhibitors of other steps of HCV replication [8].

Many pharmaceutical and biotechnology companies have initiated research and development programs to obtain better drugs for HCV. Currently there are some 40–50 compounds in clinical development, the majority of which are protease or polymerase inhibitors [9,10], the most advanced of these being the protease inhibitor telepravir (Vertex), which is in Phase 3. The first generation of direct acting HCV anti-virals is being developed as triple therapy with standard of care (SOC), namely interferon–ribavirin (INT/RBV), because single agent studies have shown the rapid emergence of resistant mutants. This experience is very similar to that of HIV therapies, suggesting that successful treatment of HCV will also require combination therapies with different mechanisms. There is a strong desire in the field to ultimately replace both interferon and ribavirin with targeted anti-virals, although this will likely take several years.

The need for combination therapy is based on the biology of the HCV and viral dynamics in the infected patient. HCV has an RNA polymerase that can synthesize transcripts to make ∼10e11 copies per day with an error rate of about 5% [11]. This gives rise to pools of virus quasi-species from which drug resistant populations can emerge rapidly. Viral kinetic studies in patients [12] suggest that there is an equilibrium between clearance of the virus by host defense mechanisms (first phase of viral clearance) and the turnover of infected hepatocytes (second phase of viral clearance) on the one hand, and the production of new viruses and infection of new hepatocytes on the other. Inhibitors of viral replication can dramatically reduce the production of new virions, but because of the pre-existence of resistant mutants in the quasi-species, cannot completely suppress it. A more effective process for viral load reduction would combine replication inhibitors with another drug that can synergize by acting on the second phase of viral clearance. Inhibitors of virus entry would achieve this by preventing the generation of new infected hepatocytes and spread of drug resistant mutant viruses.

In the quest for direct acting anti-virals, most companies have targeted viral genes involved in replication: protease and polymerase inhibitors and other non–structural proteins (e.g., NS5A) [13], while relatively little effort has been directed at host targets. This is a logical approach with evident success, but there is also an implicit assumption that targeting viral proteins will confer greater safety than targeting host cell proteins. However, one can challenge this assumption based on at least two observations: (1) the majority of drugs in use across other disease areas are directed against ‘host cell’ targets with good safety profiles and (2) drugs directed against viral replicative enzymes, e.g. protease inhibitors, often have off-target activities with accompanying safety risks, e.g. lipodystrophy for anti-retrovirals [14], and the worrisome rashes and hepatotoxicity of some HCV protease inhibitors [15]. There are some theoretical advantages to targeting host cell factors for HCV; there may be a higher barrier for the virus to generate resistance against host targets, and drugs targeting host factors are also less likely to be genotype selective, since so far all HCV genotypes seem to have by and large tapped into the same host cellular pathways. In addition, it is likely that combination therapy will continue to be required in the treatment of hepatitis C; drugs directed at host cell targets will represent a complementary mechanism of action to inhibition of viral targets. Similarly, drugs attacking steps additional to replication should synergize with replication inhibitors. Inhibition of virus entry is a well-validated approach in other viral diseases, including HIV, and is a rational strategy for HCV. Here we will provide a current perspective on HCV entry inhibitors, from scientific rationale to clinical development. Greater detail will be presented on our own compound, ITX5061, a small molecule antagonist of SR-BI, a cellular co-receptor of HCV.

## 2. The HCV Entry Process

HCV is an enveloped virus belonging to the *Flaviviridae* family of positive-strand RNA viruses (see [16] for review). HCV infects predominantly hepatocytes, although recent data indicate that neuroepithelial cells may also be a target for HCV infection [17]. The basic structure of the virion is a nucleocapsid core surrounded by a host-derived lipid bi-layer envelope containing two transmembrane glycoproteins, E1 and E2. These glycoproteins form a non-covalent, heteromeric dimer that functions to mediate virus binding and entry into host cells [5,18]. Each glycoprotein appears dependent on the other for proper folding and expression [19–21] and together may function as a class II fusion complex associated with merging of viral and host membranes [22]. The amino-terminal region of E2, termed hypervariable region 1 (HVR1), is an important determinant for antibody-mediated virus neutralization [23]. Deletion of the HVR1 domain from E2 results in an attenuated virus infection in chimpanzees [24] and reduced receptor binding of a recombinant protein [25]. HCV isolated from patient serum is heterogeneous in nature, due to virus interaction with circulating antibodies and/or lipoproteins; association with serum lipoproteins, including LDL and VLDL, appear to account for its surprisingly low buoyant density [26,27]. While the exact mechanism remains unclear, HDL appears to have an enhancing effect on HCV entry [28,29]. Overall, a picture is emerging where HCV-lipoprotein interactions appear to have important role for successful virus entry into host cells [30].

**Figure 1.**
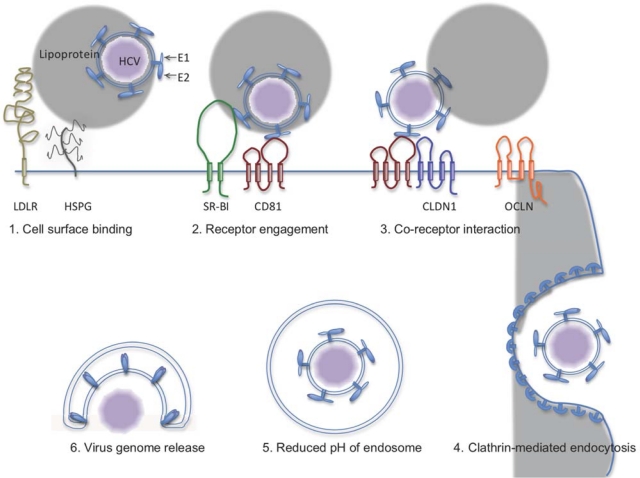
The HCV Entry Process.

Figure 1 is a simple depiction of the HCV entry process. The first step is a general association of virus with the host cell surface. Heparin sulfate proteoglycans, commonly found on the surface of many cell types, have been implicated as an initial attachment factor for HCV [31]; competition with heparin, or prior removal using heparinase, reduced viral infection [32]. Low density lipoprotein receptor (LDLR) is responsible for the binding and receptor-mediated uptake of lipoproteins into the cell, and may do the same with HCV-associated low density lipoproteins [33]. Recent studies suggest that LDLR may facilitate virus entry by binding to the ApoE ligand associated with infectious virus particles [34]. Following the binding of HCV to the cell surface, several specific entry factors participate in a complex cascade of events leading to virus internalization. The exact role of each entry factor and the precise order of events remain unclear and is an active area of investigation. Scavenger receptor class B type I (SR-BI) is the principal HDL receptor, involved in lipid and cholesterol transfer to the cell membrane, and also participates in reverse cholesterol transport through the delivery of cholesterol to catabolic pathways in the hepatocyte. It is expressed predominantly in the liver and steroidogenic tissues (for review see [35]). It was initially identified as an HCV entry factor based on its ability to bind the ectodomain of the E2 glycoprotein mediated by the HVR1 region [25]. Antibodies recognizing the extracellular domain of SR-BI are able to block HCV infection at a early step following virus binding [36]. The ability of HDL to enhance HCV infection, and the observation that mutations in SR-BI that inhibit HDL binding and lipid transfer also disrupt HCVpp infection [37], suggest a lipoprotein-related functional role for SR-BI in mediating HCV infection.

Working in concert with SR-BI is another HCV entry factor, CD81 [38]. CD81 is a ubiquitously expressed protein that belongs to a family of cell surface proteins called tetraspanins. Tetraspanins play an important role in organizing proteins within the cell membrane and may participate in membrane fusion events (for review, see [39]). CD81 was also originally identified for its ability to bind the ectodomain of E2 and this interaction is mediated by the large extracellular loop (LEL) [40]. CD81 has subsequently proven critical for entry based on the absence of infection from lack of surface expression, either naturally or through gene silencing [38,41–43], and by inhibition of entry using blocking antibodies or peptide competitors derived from the LEL region [3–7,41,44,45]. Timing of blocking antibody experiments suggests that CD81 functions at a step following virus binding and attachment [44]. Interestingly, an HCV envelope mutant with increased binding to CD81 also exhibited lesser dependence on SR-BI for virus entry, suggesting a close functional relationship of the two entry factors [46]. In addition, HCV preferentially binds to Chinese hamster ovary (CHO) cells expressing human SR-BI, compared to those expressing human CD81, suggesting SR-BI may be the first of these entry factors to interact with the virus [47].

Another four membrane-spanning molecule involved in HCV entry is claudin 1 (CLDN1), which was originally identified through an expression cloning screen in HCVpp non-permissive cells [47]. CLDN1 is highly expressed in the liver where it plays an important role in forming tight junctions at the apical surfaces of hepatocytes and biliary epithelial cells. CLDN1 appears to function at a post-binding step in HCV entry, possibly following CD81 interaction. Two amino acids (I32 and E48) located in the first extracellular domain (EC1) of CLDN1 appear critical for mediating HCVpp entry. Subsequent studies using fluorescence resonance energy transfer (FRET) experiments suggest that CLDN1 and CD81 form a co-receptor complex that is important for HCV receptor activity and that this interaction is dependent on the two critical residues in the EC1 domain of CLDN1 [48]. Most of the studies were conducted in non-polarized cell lines. In polarized hepatocytes, a majority of CLDN1 appears to be apically associated in tight junctions, but a smaller pool of protein can be detected along the baso-lateral surface [49], where virus entry most likely occurs. It is this baso-lateral CLDN1 that is thought to participate in the CD81-CLDN1 co-receptor complex formation [50].

Occludin (OCLN) is an additional tight junction protein that also mediates a post-binding step in HCV entry into host cells [51–53]. Human OCLN was also able to render mouse cells susceptible to HCVpp infection, along with expression of human CD81, suggesting that OCLN may be a species-restricting determinant of HCV infection [51]. More recently, OCLN splice variants were examined and suggested to possibly contribute to tissue tropism and outcomes of infection [54].

HCV internalization is dependent on a clathrin-mediated endocytosis [55,56]. Following internalization, HCV tracks with markers of the early endosome [57]. Acidic pH of the late endosome is thought to induce rearrangement of the E2 glycoprotein to reveal a fusion peptide that triggers viral and host membrane fusion resulting in HCV genome release [3,4,55,56,58,59].

## 3. Interventions at Virus Entry

Understanding of the above participants in the entry process for HCV infection affords numerous sites for pharmacological intervention. These include both viral and host cell targets.

### 3.1. Targeting the Viral Envelope Proteins

Viral envelope proteins have been used successfully to develop therapeutic vaccines in many viral diseases. In the case of HCV, neutralizing antibodies in acutely infected patients have been associated with virus clearance [60], and administration of broadly neutralizing antibodies recognizing E2 was able to block HCV infection in a chimeric mouse model [61]. However, the role of neutralizing antibodies in chronic HCV infection is less clear. Chronicity persists regardless of the presence of high titer, and in some cases, broadly cross-neutralizing antibodies [62]. One possible explanation is that the extreme heterogeneity of the HCV quasi-species found in patients allows the escape of variants resistant to neutralization. An alternative scenario is the recent observation that HCV transmission through cell-cell contact is refractory to neutralizing anti-E2 antibodies and patients’ sera [46]. This mode of cell-cell transmission may be an important route in chronic infection. Liang *et al*. [63] showed that in liver biopsies from HCV patients, the infected cells tend to cluster as visualized by two-photon microscopy, suggesting virus spread from single infected cells to their neighbors *in vivo*. This mechanism underlies a means of host immune evasion by the virus. These observations suggest that neutralizing antibodies directed at E2 may have a role in preventing primary infection or re-infection in liver transplant patients, but their efficacy in lowering the viral burden in chronically infected patients may be more limited.

The carbohydrate moieties of the E1 and E2 proteins have been the target of a drug discovery and development program at Migenix focused on orally active α1–glucosidase I inhibitors (see [64] for review). Both E1 and E2 have conserved N-linked glycosylation sites; the carbohydrate moieties are required for correct folding, assembly and infectivity, and they modulate the host humoral immune response to HCV infection. These inhibitors prevent the correct glycosylation of E1 and E2. MBI 3253 (CELGOSOVIR) has completed Phase 1 and 2 trials in the clinic, where it was shown to have modest efficacy as monotherapy (Phase 1b) and in combination with INT/RBV in a Phase 2b trial in non-responder patients. However, a subsequent Phase 2b trial in naïve HCV patients showed no efficacy. Migenix has since transferred its assets and operations to Biowest Therapeutics, and development of Celgosovir has apparently been suspended.

A broad-spectrum compound, arabidol (ARB), was reported to inhibit acute HCV infection, presumably by blocking virus-cell fusion [65]. One inconsistency in the results presented was that ARB had no effect on acute HCV infection if added more than 48 hours after virus input, and yet was able to suppress HCV replication in replicon cells. Furthermore, the efficacious dose of ARB for HCV inhibition (8 μg/ml) was only marginally lower than the cytotoxic dose (CC_50_ = 12.5 μg/ml), making it highly unlikely that ARB would be a development candidate.

Carbohydrate Binding Agents (CBAs) also offer possibilities for inhibiting the docking of the virion, by binding to the oligosaccharide groups of the virion glycoproteins. Two CBAs have been described: a lectin protein, Cyanovirin N, and an antimicrobial agent, Pradimicin A. Both show nanomolar potency in HCVcc and HCVpp assays, and selectivity for viral *versus* host glycans [66]. However, delivery difficulties related to molecular size would present challenges for therapeutic use. The medicinal chemistry challenges to making orally bioavailable derivatives of these ligands are considerable.

Amphipathic phosphorothioate oligonucleotides (PS-ONs) mimic the amphipathic alpha helical hinge domains of fusion proteins on many enveloped viruses, including HBV and HCV, and thereby inhibit the post-binding cell fusion/entry step. Long (>30 base) PS-ONs have been reported to inhibit HCV infection in both HCVcc and HCVpp systems with EC_50_ values in the low nanomolar range [67]. A current lead under development is REP9AC owned by Replicor, Inc. Pharmacokinetic studies showed that REP9AC has a short residence time in the blood (T_1/2_ < 1 hour) and tends to accumulate in the liver. Projected half-life in humans is more than 28 days. PS-ONs have been shown to be generally safe based on several clinical trials for other disease indications. Replicor plans to conduct proof-of-concept studies in HCV patients. The drug is administered by continuous slow infusion. Given the intense competition in the novel HCV therapeutics area, and the fact that current SOC involves subcutaneous administration of α-interferon, it would be difficult for a parenterally delivered therapeutic – whether protein, peptide, oligonucleotide or natural product – to gain wide acceptance.

### 3.2. Targeting Host Proteins Involved in Entry

As noted previously, several cellular co-receptors, namely SR-BI, CD81, CLDN 1 and OCLN, participate in the entry process, and these host cell targets could, in principle, each be exploited for targeted drug discovery. In cell-based assays, antagonism of these membrane proteins through a variety of approaches including antibodies, siRNA and small molecules, was able to inhibit infection by HCVpp or HCVcc (see also [68] for recent review). Furthermore, a monoclonal antibody against CD81 has been shown to be effective in blocking HCV infection in a chimeric mouse model with human liver implants [69]. However, drug discovery directed at co-receptor targets is still largely at the research stage. A fundamental issue in targeting a host protein is safety, and yet the safety of a given drug must be empirically determined, regardless of the known function and distribution of its target. Unlike gene knock-out systems where the function of a protein is totally ablated throughout embryogenesis and development, a drug will likely only partially inhibit an activity for a defined treatment period in adulthood (generally six months to a year for HCV therapy). It is also possible that a drug will only inhibit a select function of the protein involved in HCV infection and not other functions that the protein assumes in the cell. Furthermore, compensatory pathways may become operative to relieve the physiological defect induced by the drug. Therefore, early safety assessment in the clinic of any promising drug candidate is critical.

The most advanced program in clinical development of an HCV entry inhibitor targeting a cellular co-receptor is a small molecule SR-BI antagonist described below. SR-BI is expressed at highest levels in hepaptocytes, where it transports High Density Lipoprotein (HDL)–bound cholesterol ester for hepatocyte membrane function and for intra-hepatocyte catabolism to bile acids, and in steroidogenic tissues, such as the adrenal cortex, where it delivers cholesterol ester for synthesis of glucocorticoids [35]. As discussed earlier, SR-BI may function as a primary HCV receptor that interacts with the virus envelope proteins. It is also possible that one of the functions of SR-BI is to activate the virus for interaction with a down-stream entry factor, such as CD81. *iTherX* has developed orally bioavailable small molecule antagonists of SR-BI that inhibit HCVcc *in vitro* at subnanomolar potency. They also inhibit HCVpp infection of primary human hepatocytes containing envelope proteins derived from all known HCV genotypes [70]. Furthermore, recent data showed that the iTherX compounds are efficient in inhibiting virus spread from cell-cell contact, while this spread is refractory to neutralization to E2 antibodes [46]. Mechanistically, these compounds block the binding of soluble E2 to an hepatocyte cell line and CHO cells expressing SR-BI and the transfer of cholesterol from HDL-cholesterol into cells in an SR-BI-dependent manner [70]. Interestingly, they do not appear to inhibit LDL or VLDL mediated lipid transfer. These compounds were shown to directly bind to SR-BI, as evidenced by the specific binding of a radiolabeled analog to SR-BI expressing CHO cells, which was competed by unlabeled compounds [70].

A specific mutation in the E2 glycoprotein (G451R) has been demonstrated to enhance E2 binding to CD81 and virus infectivity, but reduce the dependency of the virus for SR-BI [71]. This mutant virus is also relatively resistant to the small molecule SR-BI antagonist compounds [70]. Interestingly, this mutation in E2 renders the virus more susceptible to neutralizing antibodies [71], and less efficient in cell-cell transmission [46]. These properties would suggest that such mutant viruses are less fit *in vivo*. It would be of interest to know if additional mutants with lesser dependence on SR-BI also exhibit such properties.

The lead compound in this series, ITX 5061, was initially developed as a p38 MAP kinase inhibitor for inflammatory disease. During preclinical and clinical studies, it was discovered unexpectedly to elevate HDL levels in animals and man. In subsequent studies the HDL elevation was shown to be due to action at SR-BI [72]. In the course of development, ITX 5061 was shown to exhibit excellent preclinical safety, including chronic toxicology studies in rats (six months) and monkeys (nine months), no genotoxicity or cardiovascular side effects and no adverse effects on coagulation parameters or elevations of liver enzymes [73]. ITX 5061 was then given to over 96 subjects in six Phase 1 studies, which yielded good safety and pharmacokinetic profiles and no elevations of liver enzymes. In addition, there was no effect on cortisol secretion pattern or response to ACTH challenge. In Phase 2 studies involving exposure to over 200 patients, no efficacy was observed in inflammatory diseases, with p38 MAP kinase as the intended target. However, a significant elevation of HDL was observed in a prospective study carried out in patients with dyslipidemia, providing *in vivo* proof of concept for activity of the drug at the SR-BI target [73]. The effect of ITX 5061 was specific to HDL elevation. There was no effect on plasma cholesterol or triglycerides, and no change in LDL or VLDL. Furthermore, there was no difference in adverse event frequency between treated and placebo control patients in all these studies. It is interesting that studies in animal models have shown that reverse cholesterol transport (RCT) can proceed in the presence of ITX 5061 [74], suggesting that the other pathways involved in RCT, such as the LDL and CETP pathways, can compensate for partial SR-BI blockade. Studies in transgenic mouse models for artherogenesis indicate that administration of ITX 5061 can actually be atheroprotective [72], suggesting a possible application of ITX 5061 and other SR-BI antagonists in treatment of cardiovascular disease. Because of the known role of SR-BI in HCV entry, iTherX tested ITX5601 in HCV entry assays and showed it to be some 500-fold more potent in inhibiting HCV entry than p38 MAP kinase activity, with a subnanomolar EC_50_ value. They also showed that HCV inhibition was not dependent on p38 MAP kinase inhibition; ITX 7650, a back-up compound in this series, has similar anti-viral potency as ITX 5061 but minimal activity MAP kinase [70]. ITX 5061 is now entering two proof-of-concept clinical studies in HCV patients. The NIH AIDS Clinical Trial Group (ACTG) will evaluate it as a monotherapy in chronically infected patients (Principal Investigator: Mark Sulkowski), the endpoints being safety in this patient population and potential efficacy (*i.e.*, reduction in viral load). The Liver Transplant Center at the University of Birmingham, UK, will initiate a trial in HCV patients receiving liver transplants (Principal Investigators: David Adams, Ian Rowe and Jane McKeating). The endpoints in this trial would be safety and kinetics of re-infection with treatment by the compound.

## 4. Random Screening for HCV Entry Inhibitors

Screening of small molecule inhibitors of virus entry has been facilitated by the development of HCV pseudovirus systems. HCVpp utilizes the unmodified HCV envelope proteins to pseudotype retroviral particles, including HIV-1. The infectivity of HCVpp depends on the co-expression of both E1 and E2 and can be neutralized by patient sera as well as monoclonal antibodies against HCV E2 protein [3,45]. Typically, HCVpp expressing a reporter gene such as luciferase is used to infect a human hepatoma cell line (e.g. Huh 7) in the absence or presence of different concentrations of a compound library [75]. A counterscreen with pseudovirus containing an unrelated viral envelope protein (e.g. VSV-G) is usually carried out in parallel to rule out non-specific effects. Compounds that efficiently inhibit HCVpp infectivity are identified as initial hits and subjected to further *in vitro* and *in vivo* characterizations. These include validation with culture HCVcc and cell viability assays in Huh7 cells and/or primary hepatocytes and mechanistic studies to identify the molecular target(s) of the inhibitors. Assessments of metabolic stability (*in vitro* microsomal assays and *in vivo* PK studies), oral bioavailability and preliminary toxicity in animals are also essential to the development process. Compounds with good drug exposure and significant oral bioavailability (F > 40%) in animals would be selected for optimization, including chemical synthesis of new analogs based on well-defined parameters of structure-activity relationship (SAR), with the aim of further improving on potency and metabolic stability and reducing toxicity.

Some companies have taken this approach to identify entry inhibitors. ITX 4520 was identified at iTherX using the HCVpp assay [76]. It showed efficacious inhibition of HCVcc infection (IC50 ∼ 1.5 nM) and desirable therapeutic index (>1000). This compound exhibits high *in vitro* metabolic stability in hepatic microsomes extracted from different species (human, rat or dog) and showed low clearance rate and good bioavailability in PK studies conducted in rat (40%) and dog (70%). Interestingly, although ITX 4520 is structurally unrelated to ITX 5061, it appears to also target SR-BI based on many of the criteria described above: it competes binding of E2 as well as a radiolabeled analog of ITX 5061 to SR-BI expressing cells, and inhibits SR-BI mediated transfer of HDL lipid into cells. It is currently a back-up candidate for iTherX’s clinical program.

Progenics Pharmaceuticals, Inc. utilized a similar approach and identified compounds that exhibited potent inhibitory activity against both HCVpp and HCVcc. In particular, PRO 206 also showed favorable pharmacokinetics and oral bioavailability [77]. However, the molecular target of PRO 206 is not known, and the compound seems to be selective for genotype 1 HCV. Recently, Progenics has suspended further preclinical development of PRO 206 in favor of other compound leads from this program [78].

## 5. Perspective

Much insight has been gained in recent years relating to the HCV life cycle, including virus entry. Therefore, it has become feasible to develop high-throughput screening assays (*i.e.,* HCVpp) that selectively address the entry step, as well as to target specific viral and cellular factors known to be involved in this process. Inhibition of virus entry has been proven effective in both acute and chronic viral infections, including HIV. HCV should be no exception. HCV entry inhibitors are expected to synergize with the many replication inhibitors now in development. Desirable properties of HCV entry inhibitors include those that pertain to anti-virals in general and those specific to entry inhibitors. The ideal HCV drug would have a large therapeutic window (high potency and low toxicity) and can be dosed once a day orally. It would work across all HCV genotypes and have a relatively high barrier to resistance. The latter may result from lesser fitness of the resistant mutant viruses relative to wild-type virus *in vivo*, and therefore, a lower prevalence of the resistant mutations in the virus population at baseline. Furthermore, since combination therapies are expected to prevail, the ideal HCV drug would exhibit additive, if not synergistic effects with other antiviral therapies, and present a distinct resistance profile. One important issue specifically relevant to HCV entry inhibition is the mode of HCV transmission. Recent studies suggest that cell-cell transmission may be a major route of virus spread in the chronically infected liver [63]. If so, it is important that an entry inhibitor can efficiently block this particular mode of transmission.

There is currently much optimism that highly potent viral replication inhibitors, either in combination with INT/RBV as first generation therapies, or as combinations of various classes of replication inhibitors, can “cure” HCV infection. However, one should not underestimate the impact of viral resistance that has already been observed with these agents. Addition of an entry inhibitor into these regimens can inhibit amplification of the mutant viruses, and expansion of the infected cell pool. Another factor is the variability of the disease courses, possibly due to heterogeneity in virus quasi species and host genetic differences in drug response, e.g. hepatic clearance differences [79]. These facts engender a need for a variety of regimens that can be tailored for subpopulations of patients for maximal benefits. In this light, development of diverse agents with different mechanisms of action may be the best course.

It should be noted that the regulatory process for clinical testing of entry inhibitors for chronic patients must take into account that the kinetics of antiviral response will differ from that of replication inhibitors. The impact on viral load will not be immediate, but will depend on the rate of turnover of the infected cell pool (second phase of viral clearance), which may be over days or weeks [12]. Currently, the FDA mandates that the first single agent study of any novel HCV therapeutic not to exceed three days due to a concern about generating resistance in patients undergoing clinical trials. This may be appropriate for replication inhibitors, but may not allow enough time for an entry inhibitor to capture the maximum antiviral effect. In the combination trials, which represent the more relevant test of efficacy, the duration of the trial should enable the effect of an entry inhibitor to be manifest.

Finally, a unique utility of entry inhibitors is in the setting of liver transplantation. HCV infection is one of the leading risk factors for liver transplantation. Unfortunately, in this population, the new graft universally gets re-infected from virus remaining in circulation, and the patients usually proceed to chronic infection with an accelerated course of disease [80,81]. Treatment with HCV entry inhibitors in the peri- and post-transplant period may reduce the rate and extent of re-infection. This could in turn reduce the morbidity/mortality and improve the outcomes for the transplant recipients.
